# Soluble factors secreted by differentiating embryonic stem cells stimulate exogenous cell proliferation and migration

**DOI:** 10.1186/scrt415

**Published:** 2014-02-24

**Authors:** Alyssa V Ngangan, James C Waring, Marissa T Cooke, Christian J Mandrycky, Todd C McDevitt

**Affiliations:** 1 The Wallace H. Coulter Department of Biomedical Engineering, Georgia Institute of Technology and Emory University, 313 Ferst Drive, Atlanta, GA 30332, USA; 2 The Parker H. Petit Institute for Bioengineering and Biosciences, Georgia Institute of Technology, 311 Ferst Drive, Atlanta, GA 30332, USA

## Abstract

**Introduction:**

Stem cells are being investigated as catalysts of tissue regeneration to either directly replace or promote cellularity lost as a result of traumatic injury or degenerative disease. In many reports, despite low numbers of stably integrated cells, the transient presence of cells delivered or recruited to sites of tissue remodeling globally benefits functional recovery. Such findings have motivated the need to determine how paracrine factors secreted from transplanted cells may be capable of positively impacting endogenous repair processes and somatic cell responses.

**Methods:**

Embryonic stem cells were differentiated as embryoid bodies (EBs) *in vitro* and media conditioned by EBs were collected at different intervals of time. Gene and protein expression analysis of several different growth factors secreted by EBs were examined by polymerase chain reaction and enzyme-linked immunosorbent assay analysis, respectively, as a function of time. The proliferation and migration of fibroblasts and endothelial cells treated with EB conditioned media was examined compared with unconditioned and growth media controls.

**Results:**

The expression of several growth factors, including bone morphogenic protein-4, insulin-like growth factors and vascular endothelial growth factor-A, increased during the course of embryonic stem cell (ESC) differentiation as EBs. Conditioned media collected from EBs at different stages of differentiation stimulated proliferation and migration of both fibroblasts and endothelial cells, based on 5-bromo-2′-deoxyuridine incorporation and transwell assays, respectively.

**Conclusions:**

Overall, these results demonstrate that differentiating ESCs express increasing amounts of various growth factors over time that altogether are capable of stimulating mitogenic and motogenic activity of exogenous cell populations.

## Introduction

Tissue damage in adult mammalian species typically results in the formation of scar tissue that prohibits the recovery of normal tissue function. Cellular infiltration, matrix deposition, angiogenesis, and remodeling events that transpire following tissue injury are stimulated to prevent further damage and preserve tissue function; however, such endogenous processes are typically insufficient to fully promote functional regeneration in adult mammals. Efforts to augment this process via transplantation of various cell populations with the ability to integrate and restore function to damaged tissues have been attempted in a number of different mammalian tissues [1-3], yet the percentage of cells that successfully engraft and persist within host tissues is typically quite low (<1%) [4-6]. However, despite the transient presence of exogenously delivered cells, persistent macroscopic functional effects and benefits have been commonly observed in a variety of different tissues.

In the absence of tissue integration, the beneficial impact of transplanted cell populations within regions of tissue damage is most probably due to the local secretion of paracrine factors produced by the exogenously delivered cells. Several studies have investigated the influence of paracrine factors secreted by cells of interest in preserving tissue integrity and function by attenuation of apoptosis and adverse remodeling [7-10]. Additionally, transplantation of exogenous stem cells and their differentiated progeny capable of secreting a potent combination of soluble factors can augment chemotaxis of host cells, including endothelial cells (ECs) and fibroblasts that are known to participate in tissue remodeling processes [11-14]. Further characterization of the soluble milieu produced by several exogenous cell types implemented in cell transplantation therapies has identified the presence of distinct secreted growth factors and chemokines known to be involved in wound healing, including vascular endothelial growth factor-A (VEGF-A), transforming growth factor beta, and fibroblast growth factor-2 (FGF-2) [15-17]. Like other stem and progenitor cells, pluripotent embryonic stem cells (ESCs) and ESC-derived cells secrete paracrine factors capable of attenuating tissue injury in a variety of settings [18-20].

Therefore, having established a paracrine mode of action for stem cells, including ESCs, there is increasing interest in the specific composition of the extracellular stem cell environment, specifically the secretome and glycome, in efforts to understand the mechanism(s) of action whereby stem cells impart therapeutic benefits [14,21-24]. Interestingly, mammalian embryos are inherently capable of endogenous functional tissue restoration and scarless wound healing, phenomena not commonly observed in most adult species, indicating that unique factors present in the embryonic environment may promote these distinctive processes [25-27]. *In vitro* differentiation of ESCs through the formation of multicellular aggregates, referred to as embryoid bodies (EBs), mimics aspects of embryogenesis, including behaviors such as the proliferation, differentiation and morphogenesis of pluripotent cells [28-30]. Thus, as ESCs differentiate within EBs to primitive cell phenotypes, the accompanying profile of molecules secreted by the cells might also be expected to reflect the dynamic morphogenic nature of the native embryonic environment [31].

The objective of this study was thus to determine the relative abundance of specific soluble factors secreted by ESCs as a function of differentiation and to examine the effects of these ESC-derived factors on exogenous cell types typically involved in wound repair and tissue remodeling, specifically fibroblast and ECs. As ESCs differentiated within EBs, the amounts of several secreted growth factors known to stimulate mitogenic and motogenic activity of somatic cells were quantified. In addition, the effects of EB-secreted factors on fibroblast and EC proliferation and migration were assayed. Overall, characterizing the identity and relative potency of growth factors secreted by differentiating ESCs on exogenous cell types may provide new insights into molecular mechanisms of how differentiated derivatives of pluripotent cells can positively impact tissue repair for acute injuries and chronic diseases and can lead to the development of a novel class of regenerative molecular therapies.

## Materials and methods

### Mouse embryonic stem cell culture

D3 murine ESCs were cultured on 0.1% gelatin-coated plates in complete medium consisting of Dulbecco’s modified Eagle’s medium (Mediatech, Manassas, VA, USA) supplemented with 15% fetal bovine serum (Hyclone, Logan, UT, USA), 2 mM l-glutamine (Mediatech), 1× non-essential amino acids (Mediatech), 100 U/ml penicillin (Mediatech), 100 mg/ml streptomycin (Mediatech), 0.25 mg/ml amphotericin (Mediatech), 0.1 mM β-mercaptoethanol (MP Biomedicals, LLC, Cleveland, OH, USA), and 10^3^ U/ml leukemia inhibitory factor (ESGRO; Millipore, Billerica, MA, USA). Cells were incubated in a humidified environment at 37°C in 5% carbon dioxide and passaged with 0.05% trypsin–ethylenediamine tetraacetic acid (Mediatech) at approximately 70% confluence. To initiate ESC differentiation, EBs were formed from a single-cell suspension of 4 × 10^6^ cells in 10 ml differentiation medium (complete medium without leukemia inhibitory factor) by spontaneous aggregation on a rotary orbital shaker. EBs were cultured in 100 mm Petri dishes on a rotary orbital shaker (Lab-Line Lab Rotator; Barnstead, Dubuque, IA, USA) held constant at 40 ± 2 rpm [32,33]. Rotary shakers were calibrated daily to ensure constant speed throughout the course of EB culture, and the medium was completely exchanged every 2 days after collecting EBs via gravity-induced sedimentation in 15 ml conical tubes.

### Collection of embryoid body conditioned medium

EBs differentiated for 4, 7, or 10 days were collected (as described above), rinsed three times with phosphate-buffered saline (PBS, pH 7.4), and cultured for an additional 2 days with a modified serum-free differentiation medium – hereafter referred to as unconditioned medium (uCM) – consisting of phenol red-free, low-glucose (2.8 mM) Dulbecco’s modified Eagle’s medium (Invitrogen, Life Technologies, Grand Island, NY, USA), supplemented with 2 mM l-glutamine, 1× non-essential amino acids, 100 U/ml penicillin, 100 mg/ml streptomycin, 0.25 mg/ml amphotericin, and 1 mg/ml bovine serum albumin (Fraction V; MP Biomedicals). After 48 hours of conditioning, EBs were collected by gravity-induced sedimentation and the EB conditioned medium (CM) (~10 ml) was transferred to a fresh conical tube. The EB-CM was centrifuged for 5 minutes at 10,000 rpm at 4°C to remove cellular debris and the supernatant fraction was transferred to a fresh 15 ml conical tube and stored at −20°C prior to further analysis.

### Embryoid body count

At each EB-CM collection time point, EBs were washed twice with PBS and resuspended in 2 ml PBS. A 200 μl sample was serially diluted (between 1:5 and 1:10) in order to reduce the density of EBs for visual counting in a 24-well plate. The final diluted volume of EBs (1 ml) was added at 200 μl per well, and the number of EBs per well (five wells) was counted. The number of diluted EBs was counted at 4× magnification using a Nikon TS100 microscope (Nikon, Inc., Melville, NY, USA) and the total number of EBs per plate was calculated based on the respective dilution factor used.

### RNA extraction and quantitative reverse transcriptase-polymerase chain reaction

RNA was extracted from undifferentiated ESCs (day 0) and from serum-free EBs on days 6, 9, and 12 of rotary culture utilizing the RNeasy Mini Kit (Qiagen, Valencia, CA, USA) and was analyzed on a NanoDrop spectrophotometer (NanoDrop, Wilmington, DE, USA) for concentration and purity from 260 nm and 280 nm absorbance readings. Complementary DNA was reverse transcribed using 1 μg total RNA in conjunction with the iScript cDNA synthesis kit (Bio-Rad, Hercules, CA, USA) on the iCycler Thermal Cycler (Bio-Rad). Quantitative reverse transcriptase-polymerase chain reaction was performed using SYBR Green with the MyIQ cycler (Bio-Rad). Primer sets were designed using Beacon Designer software (Premier Biosoft, Palo Alto, CA, USA) for pluripotency markers (Octamer-4, *Oct-4*) and differentiation markers (α-fetoprotein, *Afp*) as well as for the housekeeping gene glyceraldehyde-3-phosphate dehydrogenase (*Gapdh*). Additionally, growth factor primer sets were also designed, including bone morphogenetic protein-4 (*Bmp-4*), fibroblast growth factor-2 (*Fgf-2*), insulin-like growth factor-1 and factor-2 (*Igf-1, Igf-2*), platelet-derived growth factor-B chain (*Pdgfb*), and vascular endothelial growth factor-A (*Vegfa*). Each primer pair was independently validated with appropriate positive cell controls. Relative levels of gene expression were calculated compared with undifferentiated ESC samples and were normalized to *Gapdh* using the ΔΔC_t_ method [34]. All primer sequences used are presented in Table 1.

**Table 1:** Primer nucleotide sequences

**Primer**	**Nucleotide sequence (5′ to 3′)**	
*Gapdh*	Forward	GCC TTC CGT GTT CCT ACC
	Reverse	GCC TGC TTC ACC ACC TTC
*Oct-4*	Forward	CCG TGT GAG GTG GAG TCT GGA G
	Reverse	GCG ATG TGA GTG ATC TGC TGT AGG
*Afp*	Forward	CAC ACC CGC TTC CCT CAT CC
	Reverse	TTC TTC TCC GTC ACG CAC TGG
*Bmp-4*	Forward	CTG GCC CGG AAG CTA GGT GAG TT
	Reverse	GAG GGC CAG AGA CTG GAT CGC
*Fgf-2*	Forward	AGC GAC CCA CAC GTC AAA CTA C
	Reverse	CAG CCG TCC ATC TTC CTT CAT A
*Igf-1*	Forward	TCC GCC AGG TTG CCT CTA G
	Reverse	GGA AGC AGG TGG ATG GTC AG
*Igf-2*	Forward	TCA AAG AGT TCA GAG AGG
	Reverse	CAA CCA TCA GTG AAT CAA A
*Pdgfb*	Forward	ATC GCC GAG TGC AAG ACG CG
	Reverse	AAG CAC CAT TGG CCG TCC GA
*Vegfa*	Forward	TGC ACC CAC GAC AGA AGG
	Reverse	GCA CAC AGG ACG GCT TGA

Forward and reverse primer nucleotide sequences for the polymerase chain reaction amplification of glyceraldehyde-3-phosphate dehydrogenase (*Gapdh*), Octamer-4 (*Oct-4*), α-fetoprotein (*Afp*), bone morphogenetic protein-4 (*Bmp-4*), fibroblast growth factor-2 (*Fgf-2*), insulin-like growth factor-1 and factor-2 (*Igf-1*, *Igf-2*), platelet-derived growth factor-B chain (*Pdgf-b*), and vascular endothelial growth factor-A (*Vegfa*). All reverse transcriptase-polymerase chain reactions performed with an annealing temperature of 58°C.

### Embryoid body conditioned medium protein quantification

The total protein content of EB-CM was analyzed using a bicinchoninic acid assay kit (Thermo Scientific, Pittsburgh, PA, USA). According to the manufacturer’s protocol, a 25 μl sample was incubated with 200 μl bicinchoninic acid solution for 30 minutes. Absorbance readings were taken at 562 nm using a Molecular Devices SpectraMax M2^e^ microplate reader and SoftMax Pro v5 microplate data acquisition software (Molecular Devices, Sunnyvale, CA, USA). The absorbance readings of the EB-CM samples were compared against a standard curve (0 to 2,000 μg/ml) generated using bovine serum albumin in order to calculate absolute protein concentrations. To determine the amount of protein secreted by the EBs during conditioning, the protein content of uCM was subtracted from all of the EB-CM samples.

Enzyme-linked immunosorbent assay kits were used to quantify the amount of proteins of interest contained within the EB-CM, specifically BMP-4, IGF-2 and VEGF-A (DuoSet; R&D Systems, Minneapolis, MN, USA). Briefly, capture antibody was adsorbed onto a MaxiSorp™ Immuno 96-well plate (Nunc, Thermo Scientific), followed by a blocking step, incubation with 100 μl sample, and binding of analyte to a biotinylated detection antibody. The concentrations of capture and detection antibodies used were dictated by the DuoSet protocol for each protein: 2 μg/ml and 1 μg/ml for BMP-4, 4 μg/ml and 200 ng/ml for IGF-2, and 400 ng/ml and 100 ng/ml for VEGF-A. uCM was used as the diluent for the standard curve samples. The amount of analyte was assessed using the colorimetric reaction of peroxidase and tetramethylbenzidine at an absorbance reading of 450 nm. The absorbance values for each EB-CM sample were compared with the standard curve to establish the protein analyte content, which was normalized to the EB number and reported as picograms of protein per 1,000 EBs.

### Proliferation assay

NIH/3T3 fibroblasts were cultured on tissue culture dishes in 3T3 growth medium containing Dulbecco’s modified Eagle’s medium supplemented with 10% bovine growth serum, 4 mM l-glutamine, 100 U/ml penicillin, 100 μg/ml streptomycin, and 0.25 μg/ml amphotericin. Human umbilical vein endothelial cells (HUVECs) were cultured on gelatin-coated tissue culture dishes in EC growth medium, consisting of MCDB131 basal medium supplemented with 5% fetal bovine serum, 100 μg/ml streptomycin, 0.25 μg/ml amphotericin, 2 mM l-glutamine, 1 mg/ml hydrocortisone (Sigma, St. Louis, MO, USA), 2 ng/ml FGF-2 (PeproTech, Princeton, NJ, USA), 10 ng/ml epidermal growth factor (Gibco, Life Technologies), 2 ng/ml IGF-1 (Gibco), 1 ng/ml VEGF-A (Sigma), and 50 mg/ml ascorbic acid (Sigma).

For proliferation studies, NIH/3T3 fibroblasts and HUVECs were cultured to approximately 50% confluence in two-chamber glass slides (BD Bioscience, San Jose, CA, USA), at which point growth media were removed and the cells were rinsed three times in PBS. Cells were then starved overnight in low-serum media, followed by an 18-hour pulse with 10 μM 5-bromo-2′-deoxyuridine (BrdU) in EB-CM from different time points as well as control media. After the 18-hour pulse, cells were rinsed with PBS and fixed in 70% ethanol with 2.3 M HCl for 10 minutes at room temperature, rinsed in PBS three times, and incubated with mouse anti-BrdU primary antibody (Molecular Probes, Life Technologies) for 1 hour at room temperature. Following primary antibody incubation, the cells were incubated with donkey anti-mouse secondary antibody conjugated to AlexaFluor®488 (Invitrogen) for 2 hours at room temperature, counterstained with 10 μg/ml Hoechst 33258 (Sigma), and then coverslipped using GelMount (Electron Microscopy Sciences, Hatfield, PA, USA).

Stained slides were imaged using a Nikon 80i upright microscope with a SPOTFlex digital camera and SPOT Advanced Software (Nikon) using both the fluorescein isothiocyanate (FITC) and 4′,6-diamidino-2-phenylindole (DAPI) channels. Nuclei counts were performed on 4′,6-diamidino-2-phenylindole and fluorescein isothiocyanate filtered images to calculate the percentage of BrdU-positive nuclei (fluorescein isothiocyanate) per field (six fields per chamber) using the Count Nuclei module within the MetaMorph software package (Molecular Devices).

### Transwell migration assays

For migration studies, NIH/3T3 fibroblasts and HUVECs were each grown to ~80% confluence, inactivated with 10 μg/ml mitomycin-C in serum-free media (2 hours at 37°C in 5% carbon dioxide) and cultured overnight in their respective growth media. Immediately prior to the initiation of transwell migration studies, cells were also fluorescently labeled in serum-free media with 1 μM CellTracker® Green (Molecular Probes) for 20 minutes at 37°C, followed by a 30-minute incubation in growth media.

HTS Fluroblok transwell culture inserts (24-well, 8 mm pore size; Falcon, BD Biosciences) were prepared with a 300 μl single-cell suspension (7.5 × 10^4^ 3T3 cells or 1 × 10^5^ HUVECs) in growth media (loaded in the top chamber). The bottom of the transwell chamber was filled with 800 μl EB-CM sample or control media; cells’ respective growth media were used as positive controls, while uCM served as a negative control. Transwell culture plates were incubated for 24 hours at 37°C and 5% humidity, and fluorescent microscopy images of the bottom of wells were taken every 6 hours using a Nikon TE 2000 inverted microscope equipped with a SpotFLEX digital camera and SPOT software (Nikon). The number of cells that migrated through the porous membrane were counted for each transwell sample (three fields of view) using ImageJ (open source program, NIH, Bethesda, MD, USA) and normalized per square millimeter.

### Statistical analysis

All experimental samples were analyzed with *n* = 6, with data presented as mean ± standard error. Statistical significance was determined using SYSTAT 12 (SYSTAT Software, Inc., San Jose, CA, USA) employing an analysis of variance test with *post-hoc* Tukey analysis to determine significance (*P* < 0.05).

## Results

### Embryoid body differentiation

**Figure 1:**
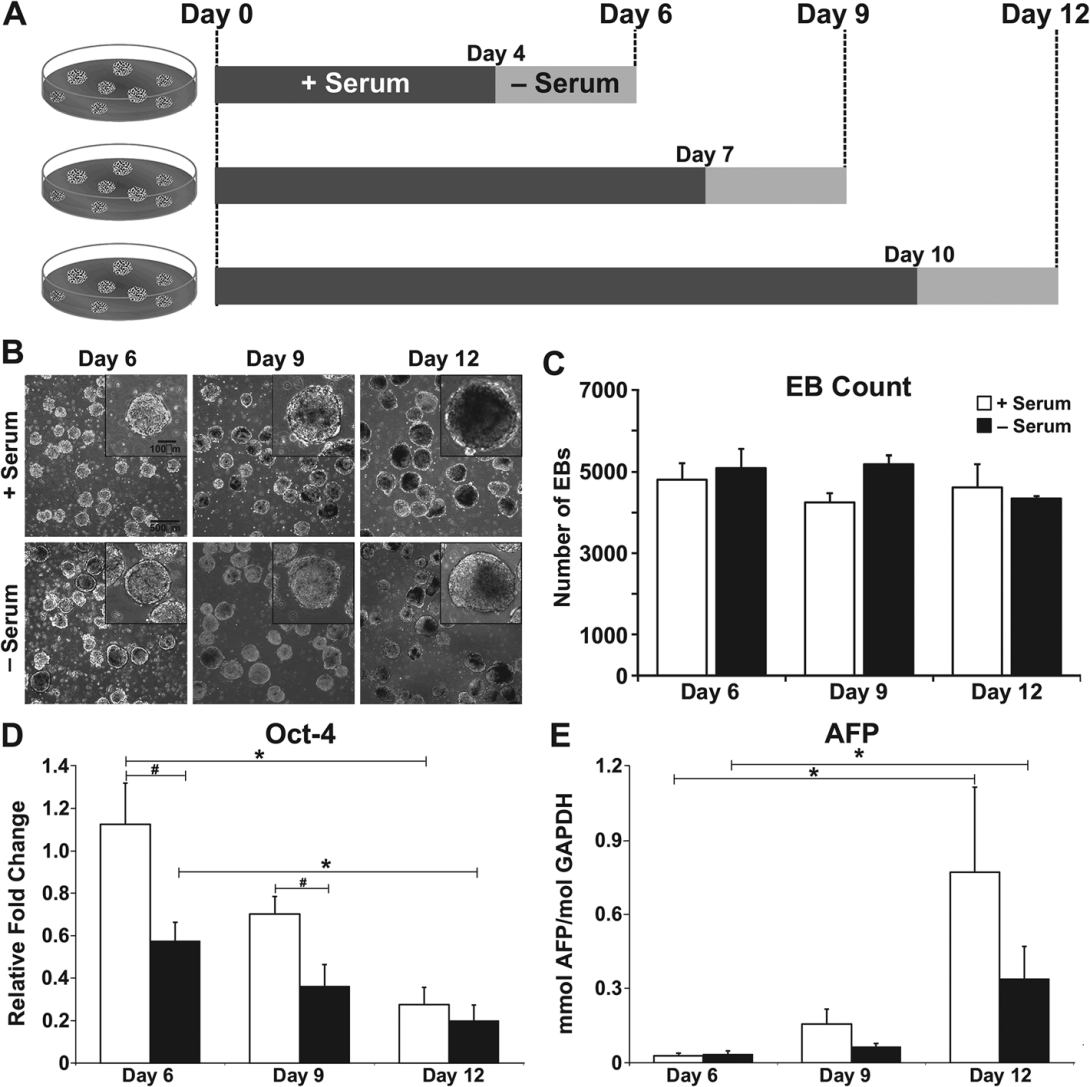
**Embryoid body differentiation and conditioned media collection. (A)** Embryoid bodies (EBs) were cultured on a rotary orbital shaker at 40 rpm for 4, 7, and 10 days in serum-containing media, at which point the EBs were switched to serum-free media and cultured for an additional 2 days. After 6, 9, or 12 days of differentiation, EB conditioned media were collected for further analysis. **(B)** EBs cultured with (top row) or without (bottom row) serum were similar in size, shape, and morphology at each of the time points examined. Scale bars = 500 μm (panel) and 100 μm (inset). **(C)** The number of EBs remained relatively constant per culture (~5 × 10^3^ EBs/plate) independent of the culture media used. **(D, E)** Similar EB differentiation in both media conditions was confirmed by polymerase chain reaction analysis, which revealed a significant decrease in the pluripotent marker *Oct-4* by day 12 compared with day 6 and a significant increase in endoderm marker *Afp* by day 12 compared with day 6 for EBs cultured under both conditions. Results indicate the mean ± standard error (*n* = 6). Analysis of variance: *P < 0.05; Student *t* test: **#***t* < 0.05. AFP, α-fetoprotein; GAPDH, glyceraldehyde-3-phosphate dehydrogenase; Oct-4, Octamer-4.

Preliminary studies indicated that EBs failed to initially form well in serum-free media and did not survive for more than 48 hours after being switched to serum-free conditions, especially at earlier stages of differentiation (that is, days 4 or 7). A protocol for forming and maintaining EBs in serum-containing media before switching to serum-free media for conditioning was thus used for all subsequent studies (Figure 1A). EBs cultured for up to 48 hours with serum-free medium at different stages of differentiation were morphologically similar to their cohorts that remained in serum-containing media for the same period of time (Figure 1B). As is normally observed, EBs increased in size during the course of differentiation (~250 μm diameter at day 6 compared with ~500 μm at day 12), but there were no observed differences in the size of EBs cultured in serum-free media for 2 days compared with EBs in serum-containing media (Figure 1B). The number of EBs per 100 mm Petri dish (~5 × 10^3^ EBs) also did not vary significantly over time in serum-containing or serum-free media, indicating that serum-free culture for 48 hours did not adversely affect maintenance of EBs in culture and that similar numbers of EBs were present during serum-free media conditioning (Figure 1C). Most importantly, the expression of pluripotent markers decreased over the course of differentiation, whereas various markers for differentiated lineage were increasingly expressed with EB culture time. For example, *Oct-4*, a pluripotent transcription factor, exhibited a similar pattern of decreasing expression for both EB culture conditions as differentiation proceeded (Figure 1D). At early time points, days 6 and 9, *Oct-4* gene expression levels were significantly decreased between serum and serum-free EB cultures (*P* ≤ 0.027), but by day 12 *Oct-4* gene expression was no longer significantly different between these groups although both were decreased compared with their respective day 6 EB cultures (*P* ≤ 0.025). In addition, expression of differentiated markers such as *Afp*, a marker of primitive and definitive endoderm, was increased by day 12 in both culture conditions compared with day 6 (*P* ≤ 0.034), yet between both EB culture conditions the *Afp* expression at each time point examined was similar (Figure 1E). Thus, switching EB cultures to serum-free medium for up to 48 hours did not significantly alter the differentiation of ESCs based on morphological and phenotypic analysis.

### Growth factor gene expression analysis

**Figure 2:**
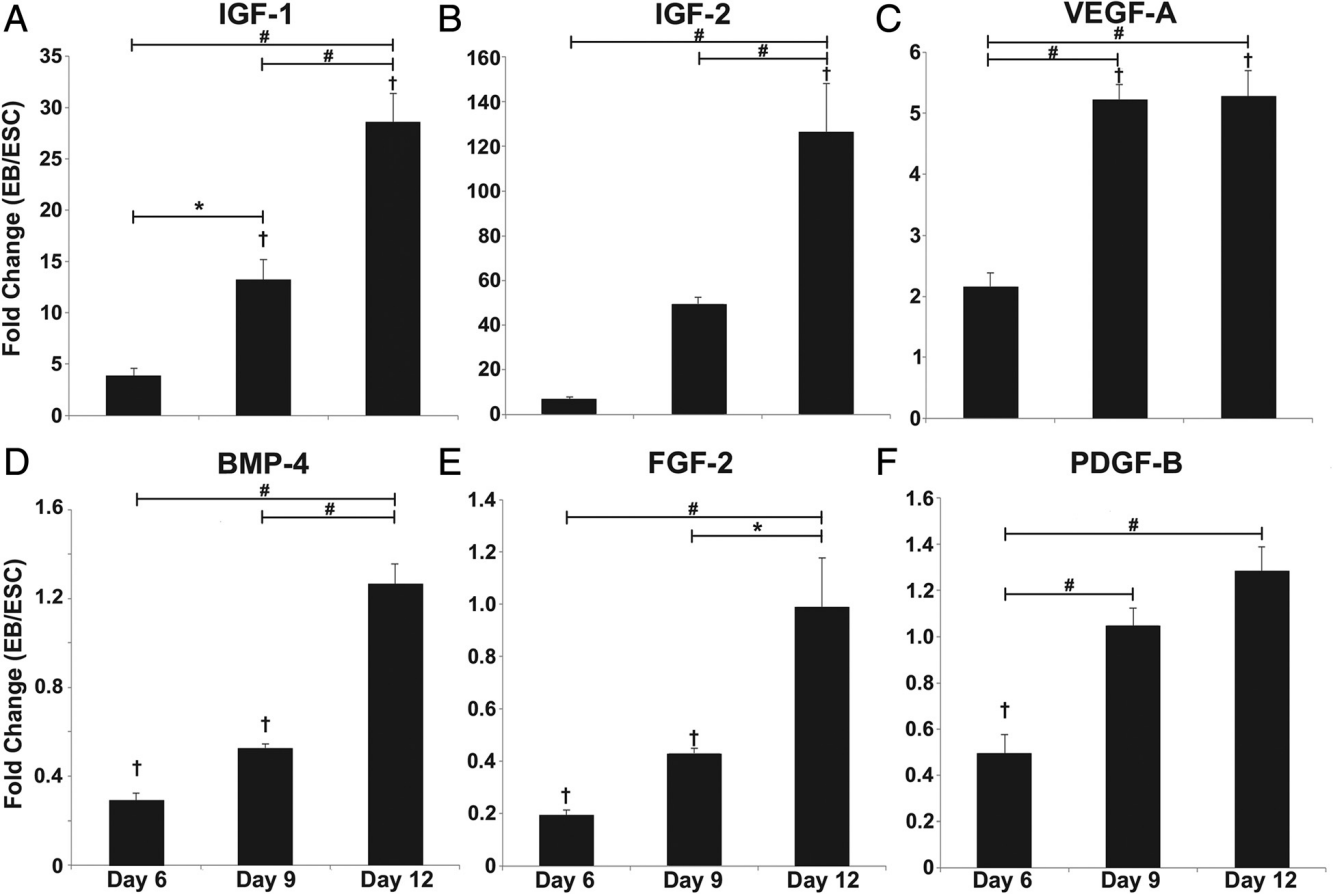
**Growth factor gene expression by differentiating embryoid bodies.** Polymerase chain reaction analysis indicated that the gene expression of several growth factors increased over time during embryonic stem cell (ESC) differentiation within embryoid bodies (EBs) **(A, B, C)** while others decreased relative to ESCs during early differentiation and then increased as differentiation progressed **(D, E, F)**. The relative values of expression are reported as the relative fold-changes by EBs compared with the starting population of undifferentiated ESCs. Results indicate the mean ± standard error (*n* = 6). Analysis of variance: *P < 0.05, #*P* < 0.001, and †*P* < 0.05 compared with ESCs. BMP-4, bone morphogenic protein; FGF-2, fibroblast growth factor-2; IGF, insulin-like growth factor; PDGF-B, platelet-derived growth factor-B; VEGF-A, vascular endothelial growth factor-A.

Overall, gene expression changes for several growth factors expressed by EBs generally increased as differentiation proceeded, measured by quantitative reverse transcriptase-polymerase chain reaction. The largest changes in growth factor gene expression compared with ESCs were observed for *Igf-1* and *Igf-2*: *Igf-1* expression increased ~13-fold and ~29-fold by day 9 (*P* = 0.009) and day 12 (*P* = 6.58 × 10^−6^) (Figure 2A), and *Igf-2* expression increased ~126-fold by day 12 (*P* ≤ 7.43 × 10^−5^) (Figure 2B). *Vegfa* gene expression was modest relative to *Igf*, yet demonstrated significant increases at day 9 (~5-fold) and day 12 (~5-fold) compared with both ESCs (*P* ≤ 6.37 × 10^−6^) and day 6 EBs (*P* ≤ 9.19 × 10^−6^) (Figure 2C). In some cases, gene expression of several growth factors was initially reduced during early EB differentiation relative to ESCs, but then increased as EB differentiation progressed. The levels of *Bmp-4*, *Fgf-2* and *Pdgfb* gene expression were decreased by 3.3-fold, twofold, and 50-fold, respectively, at day 6 compared with ESCs (*P* ≤ 0.015) (Figure 2D,E,F). At day 9, levels of *Bmp-4* and *Fgf-2* expression were similar to day 6 levels, about one-half that of ESCs (*P* = 4 × 10^−3^), while *Pdgfb* expression was increased compared with day 6 (*P* ≤ 0.001). By day 12, all three genes (*Bmp-4*, *Fgf-2*, and *Pdgfb*) were increased in expression compared with day 6 EBs (*P* ≤ 0.001), but were similar to undifferentiated ESC levels. Despite the specific differences in individual growth factor gene expression patterns, all of the aforementioned growth factors generally increased as ESC differentiation transpired.

### Protein characterization of embryoid body conditioned medium

**Figure 3:**
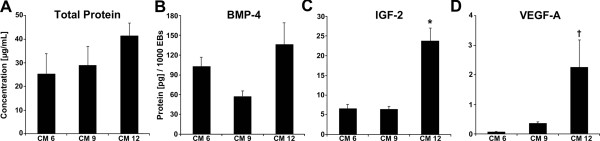
**Protein secretion by embryoid bodies into conditioned media. (A)** Total protein content of the conditioned medium (CM) collected at each time point examined as determined by bicinchoninic acid did not significantly vary over time. **(B)** Enzyme-linked immunosorbent assay results revealed that BMP-4 protein content was similar as embryoid body (EB) differentiation progressed over time. **(C, D)** Between EB-CMs, IGF-2 protein increased significantly over time in CM at day 12 (CM 12) compared with CM at day 6 (CM 6) and at day 9 (CM 9); VEGF-A increased in CM 12 with significance compared with only CM 6. Results indicate the mean ± standard error (*n* = 6). Analysis of variance: *P < 0.05 compared with CM 6 and CM 9, †*P* < 0.05 compared with CM 6. BMP-4, bone morphogenic protein; IGF, insulin-like growth factor; VEGF-A, vascular endothelial growth factor-A.

Over the course of EB differentiation, the total amount of secreted protein content in the conditioned media did not vary significantly, with results from bicinchoninic acid analysis measuring consistently within a range of 150 to 225 μg/ml (Figure 3A). Based on SDS-PAGE analysis, minor differences were apparent in the molecular content of EB-CM from different stages of differentiation, especially species between 10 and 20 kDa, corresponding to the molecular weight range of many growth factors (Additional file 1). Consistent with PCR analysis, Enzyme-linked immunosorbent assays indicated notable changes in the quantities of specific growth factor proteins in EB-CM that exhibited large fold-changes in gene expression, namely BMP-4 and IGF-2. VEGF-A was also examined due to significant gene expression changes during EB differentiation, as well as its previous examination in several stem cell conditioned media studies [15-17]. Generally, the secretion of IGF-2 and VEGF-A proteins both increased as EB differentiation progressed, corresponding to similar increases in gene expression, whereas BMP-4 content in CM did not change significantly over time (Figure 3B,C,D). Specifically, EBs at earlier stages of differentiation secreted similar amounts of IGF-2 in CM at day 6 and at day 9 (CM6, CM 9; ~6 pg/1,000 EBs), but at later stages of differentiation, day 12 EBs produced more IGF-2 (~24 pg/1,000 EBs) compared with both earlier time points. VEGF-A secretion by EBs followed a similar trend, as the protein level was increased in CM at day 12 (CM 12; 2.3 pg/1,000 EBs) compared with CM 6 (0.1 pg/1,000 EBs; *P* = 0.029). Thus, despite the similar amount of total protein secreted by EBs at different stages of differentiation, specific growth factors were differentially expressed over time.

### Mitogenic activity of embryoid body conditioned medium

**Figure 4:**
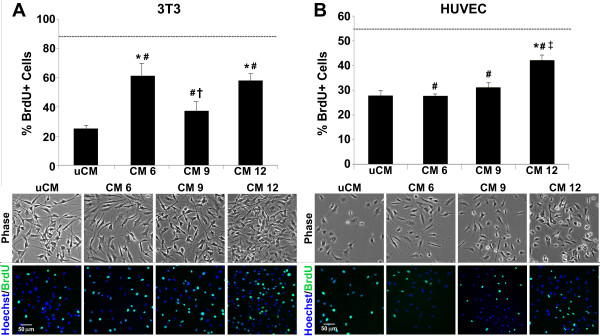
**Proliferation of exogenous cell types treated with embryoid body conditioned medium. (A)** Conditioned medium at day 6 (CM 6) and at day 12 (CM 12) increased fibroblast proliferation compared with unconditioned medium (uCM). CM at day 9 (CM 9) elicited less mitogenic response compared with CM 6 and CM 12. **(B)** Early time points of embryoid body (EB)-CM did not significantly induce endothelial cell proliferation compared with uCM, whereas CM 12 exhibited significantly greater proliferative response than all other EB-CMs and uCM. Results indicate the mean ± standard error (*n* = 6). Top row: phase images, scale bar = 50 μm. Bottom row: fluorescent images, blue = Hoechst stain, green = 5-bromo-2′-deoxyuridine (BrdU)-positive cells, scale bar = 50 μm. Dashed line, % BrdU-positive cells stimulated by the respective cell type’s growth media. Analysis of variance: *P < 0.05 compared with uCM; #*P* < 0.05 compared with growth media; †*P* < 0.05 compared with CM 6 and CM 12; ‡*P* < 0.05 compared with CM 6 and CM 9. HUVEC, human umbilical vein endothelial cell.

Following molecular composition analysis of EB-CM, somatic cells were treated with EB-CM to assess the mitogenicity of EB secreted factors. As expected, fibroblast growth medium (containing 10% bovine growth serum) stimulated BrdU incorporation of approximately 90% of the cells. CM 6 and CM 12 induced significantly more incorporation of BrdU (~60%) by fibroblasts compared with uCM (~25%, *P* ≤ 4.54 × 10^−4^), while CM 9 stimulated significantly less proliferation (~37% of fibroblasts) compared with CM 6 and CM 12 (Figure 4A). The influence of EB-secreted factors on proliferation of a more tissue-specific cell type (ECs) was additionally examined. In response to EC growth media, ~55% of ECs were BrdU-positive, whereas ECs treated with CM 6 and CM 9 resulted in ~30% BrdU-positive cells, similar to the percentage stimulated by uCM (Figure 4B). However, CM 12 stimulated significantly more HUVEC DNA synthesis (~40%) compared with the other EB-CM samples (*P* ≤ 0.002) and uCM (*P* = 1.43 × 10^−4^). All together, these results indicate that EB-CM generally exerted a more potent effect on fibroblast proliferation, and EB-CM collected from more differentiated EBs enhanced the proliferation of ECs.

### Motogenic potential of embryoid body conditioned medium

**Figure 5:**
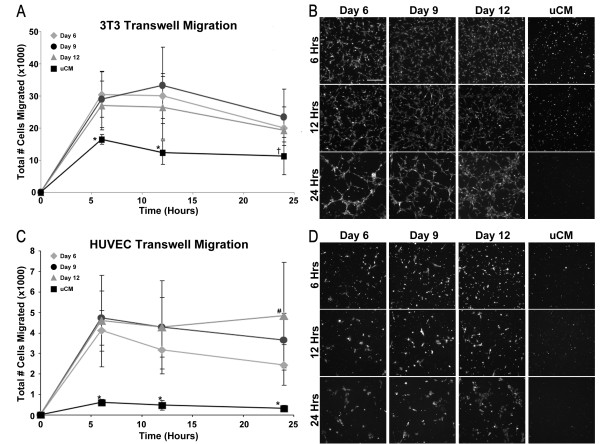
**Migration of exogenous cell types in response to embryoid body conditioned medium. (A)** Fibroblast migration was stimulated by embryoid body (EB) conditioned medium (CM) collected from different days of differentiation compared with unconditioned medium (uCM) up to 12 hours, but at 24 hours only CM at day 9 (CM 9) induced greater cell migration compared with uCM. **(B)** After 24 hours, EB-CM promoted the formation of fibroblast network structures compared with cells treated with uCM, which remained more as single cells. **(C)** Endothelial cell migration was stimulated by all of the EB-CM compositions compared with uCM. By 24 hours, CM at day 12 (CM 12) induced significantly more cells to migrate compared with CM at day 6 (CM 6) and CM 9. **(D)** After 24 hours, endothelial cells treated with EB-CM prompted cells to cluster whereas the uCM did not influence the organization of endothelial cells. Results indicate the mean ± standard error (*n* = 6). Analysis of variance: *P < 0.05 compared with all EB-CM time points; #*P* < 0.05 compared with CM 6 and CM 9; †*P <* 0.05 compared with CM 9. HUVEC, human umbilical vein endothelial cell.

The chemotactic response of somatic cells to factors secreted by differentiating EBs was assessed using a transwell assay (Figure 5). Quantitative fluorescent spectroscopy analysis showed that all CM samples stimulated more fibroblasts (~27,000 to 30,000 cells) to migrate after 6 and 12 hours of incubation compared with uCM (~16,000 cells, *P* < 0.05; Figure 5A), but by 24 hours of treatment only CM 9 stimulated more cells than uCM (~23,000 total cells, *P* = 0.008). Further assessment of the images taken at 24 hours illustrated 3T3 arrangement in clusters with extended processes with EB-CM treatment compared with single rounded cells sparsely scattered in transwells containing uCM (Figure 5B). The results from the transwell assay were similar to those obtained from an independent fibroblast scratch wound assay, where CM 9 stimulated significant wound closure by 24 hours compared with uCM (Additional file 2). Overall, the EB-CM increased fibroblast migration and modulated the morphology of both individual cells and cell clusters.

To further evaluate the motogenic effects of EB-CM on other cell types important in tissue remodeling, ECs were also examined. Over the entire 24-hour period of the migration experiment, each of the EB-CM samples stimulated more HUVEC migration than uCM (*P* < 0.05, Figure 5C). Comparisons between each of the EB-CM samples at 6-hour and 12-hour time points indicated similar motogenic induction since the numbers of migrated cells (~4,000 to 4,800 cells) were not significantly different. However, by 24 hours the number of migrated cells induced by CM 12 was greater than that by CM 6 and CM 9, due to the decreased migration between 12 and 24 hours in cells exposed to CM from earlier time points (Figure 5C). Fluorescent images illustrate that HUVECs in EB-CM were organized as clusters of larger, more spread cells, compared with the more round and individually distinct HUVECs in uCM. Additional examination of EC morphology after 24 hours revealed that CM 12 treatment yielded cells arranged in large clusters, while cells in CM 6 and CM 9 grouped in smaller clusters (Figure 5D).

Despite similar concentrations of total secreted protein in EB conditioned media collected at all time points of differentiation, different cell types responded quite differently to the ESC secreted factors. The number of 3T3 fibroblasts that migrated was about 10-fold higher than the number of migrating HUVECs throughout the transwell studies despite inoculation with similar numbers of starting cells (7.5 × 10^4^ 3 T3 cells, 1 × 10^5^ HUVECs). Overall, the results of the migration assays revealed that EB-CM collected at different stages of EB differentiation contained chemotactic factors capable of stimulating the migration of different cell types.

## Discussion

This study examined several of the growth factors that were differentially expressed by differentiating ESCs and the impact of this complex mixture of secreted factors on different somatic cell types found in most tissues. ESC transplantation has been widely studied for the repopulation of tissues undergoing remodeling; however, the number of ESCs that integrate and differentiate within the host tissue remains quite low [35,36]. Several studies have examined the potency of ESC-secreted molecules and their impact on cell and tissue physiology, including inhibition of apoptosis, rescue of congenital defects, and cardioprotection following myocardial infarction [18-20], as well as suppressing seizure activity [37]. Previous reports of ESC-secreted factors generally examined paracrine actions of undifferentiated ESCs in monolayer culture [21,38]. However, upon transplantation *in vivo*, the molecular environment within the injured tissue is more probably prone to induce differentiation rather than to maintain pluripotency, thus modulating the paracrine factors released in a manner more analogous to EB differentiation.

In this study, changes in secreted factor production coincident with EB differentiation were analyzed for protein content and bioactivity, specifically motogenicity and mitogenicity of fibroblasts and ECs. The gene expression of EBs and protein profiles of EB conditioned media demonstrated differential representation of several growth factors as differentiation progressed. Analysis of *Bmp-4* gene expression exhibited significant increases over the time course of differentiation by day 12 EBs, yet the amount of BMP-4 protein present in CM from each stage of differentiation was similar. On the other hand, the gene expression of other growth factors (particularly *Igf-2* and *Vegf-a*) increased steadily as ESC differentiation progressed and yielded increased protein content in CM 9 and CM 12. The effects of the differential expression and secretion of proteins by the differentiating ESCs was further investigated by examining varied cell responses during proliferation and migration experiments comparing EB-CM collected at different days. Overall, fibroblast migration was significantly stimulated by all CM groups up to 12 hours, with the most effect seen in the first 6 hours, followed by a plateau, and then decreasing for the remainder of the time course, when only CM 9 induced significantly more migration than uCM. While enhancing migration, CM 9 did not stimulate fibroblast proliferation compared with CM 6 and CM 12, conditions that elicited roughly equivalent levels of proliferation (Figures 4A and 5B). Distinct fibroblast behavior in response to CM 9 demonstrates the unique combination of morphogens secreted by EBs after 9 days of differentiation – a secretome that stimulates fibroblast migration, but inhibits proliferation; whereas day 6 and day 12 EBs yield secreted factors that promoted fibroblast mitogenicity. Such a change in the promotion of different cellular processes may reflect the different stages of embryogenesis mimicked by ESCs undergoing differentiation within EBs. Undifferentiated ESCs typically proliferate at a high rate, which helps establish a critical mass for EB formation. As EB differentiation proceeds, typically between 7 and 10 days, a sizable portion of the cell population in the presence of serum proteins undergoes an epithelial-to-mesenchymal transition, which is characterized by the shift of nonmigratory epithelial cells to more migratory mesenchymal phenotypes, similar to post-gastrulation embryonic development [39,40]. After epithelial-to-mesenchymal transition occurs, cells comprising EBs continue to differentiate into more specific phenotypes and proliferation rates generally increase yet again.

The response of ECs to EB-CM was different from that of fibroblasts, which is probably to be expected based on the phenotypic differences between these two cell populations. While the overall patterns of EC migration in response to the EB-CM treatments largely followed those of fibroblasts, and were significant compared with uCM during the initial 12 hours of treatment, the number of migrating ECs increased in response to CM 12 between hours 12 and 24 (Figure 5D). The proliferative capacity of ECs was also increased in response to CM 12 compared with CM 6 and CM 9 (Figure 4B). Overall, the complex mixture of morphogens secreted by day 12 EBs was more mitogenic and motogenic relative to factors secreted by day 6 and day 9 EBs. This complex combination of factors is analogous to the secretome produced by EBs as they mature, typically from days 10 to 14 of culture, when progenitor cells differentiate towards more lineage-restricted cell types [31].

The growth factors secreted by EBs that were examined in this study have been previously implicated in attenuation of tissue damage and wound healing, including regeneration of vasculature and bone. The persistence of BMP-4 protein in the EB-CM from days 6 through 12 suggests the importance of this molecule throughout early stages of ESC differentiation, specifically within the mesoderm lineage, where BMP-4 has been implicated in enhancing the formation of vascular networks produced by mammalian ESC-derived EBs [41,42]. VEGF-A is a well-documented growth factor capable of promoting angiogenesis in ischemic tissues, where it is necessary to promote an environment conducive to the effective delivery of cells and nutrients required for stimulation of tissue repair [43-45]. Many studies have demonstrated the function of IGF-2 in the growth and differentiation of skeletal muscle and bone tissue [44,46-48]. Moreover, IGFs and BMP-4 can increase VEGF expression to synergistically stimulate tissue repair, and BMP-4 in concert with VEGF treatment enhances bone formation and healing compared with single-factor treatment [49]. Similarly, IGF-2 and VEGF together enhance angiogenesis by promoting the homing of endothelial progenitor cells [50], and the combination of IGF-1 and VEGF can target multiple regenerative processes: angiogenesis, reinnervation, and myogenesis [51-53]. The increased production of these growth factors by EBs at days 9 and 12 suggests that early differentiating ESCs secrete factors that can positively affect tissue remodeling by stimulating regeneration through induction of angiogenesis, coincident with other morphogenic events.

## Conclusions

Overall, this study demonstrates that factors secreted by ESCs undergoing morphogenic differentiation as EBs are capable of inducing proliferation and migration of exogenous somatic cell types, such as fibroblasts and ECs. The complex mixture of ESC-secreted molecules contains a number of different growth factors and morphogens whose relative abundance varies as a function of EB differentiation. Not surprisingly, the morphogenic events known to be associated with EB differentiation, including cell proliferation and migration, are reflected by the endogenous expression of factors that comprise the secreted soluble milieu. Critically analyzing the composition and bioactivity of molecules secreted by differentiating ESCs thus not only provides insights into the paracrine environment within the EB, but also suggests potential trophic mechanisms whereby differentiated progeny from ESCs can beneficially impact exogenous cells commonly involved in tissue remodeling and regeneration processes.

## Abbreviations

AFP: α-fetoprotein; BMP4: bone morphogenic protein; BrdU: 5-bromo-2′-deoxyuridine; CM: conditioned medium; EB: embryoid body; EC: endothelial cell; ESC: embryonic stem cell; FGF-2: fibroblast growth factor-2; GAPDH: glyceraldehyde-3-phosphate dehydrogenase; HUVEC: human umbilical vein endothelial cell; IGF: insulin-like growth factor; Oct-4: Octamer-4; PBS: phosphate-buffered saline; PDGF-B: platelet-derived growth factor-B; uCM: unconditioned medium; VEGF-A: vascular endothelial growth factor-A.

## Competing interests

The authors declare that they have no competing interests.

## Authors’ contributions

AVN and TCM conceived of the study and designed the experiments. AVN, JCW, MTC, and CJM performed the experiments. AVN, JCW, MTC, CJM and TCM analyzed the data. AVN, MTC and TCM wrote the manuscript. All authors read and approved the final manuscript.

## Supplementary Material

Additional file 1 Figure S1 showing SDS-PAGE of EB-CM samples: image of SDS-PAGE gel. Click here for file

Additional file 2 Figure S2 showing the scratch wound migration assay: (A) phase image and (B) graph of cell migration. Click here for file
