# Supplementary material for: Soluble factors secreted by differentiating embryonic stem cells stimulate exogenous cell proliferation and migration
Source: Stem Cell Res Ther. 2014 Feb 24;5(1):26. doi: 10.1186/scrt415 (PMC4055104; doi:10.1186/scrt415)
Supplement: Additional file 1 — Figure S1 showing SDS-PAGE of EB-CM samples: image of SDS-PAGE gel. [file scrt415-S1.doc]

**Additional file Figures**

**Additional file 1: Figure S1.**


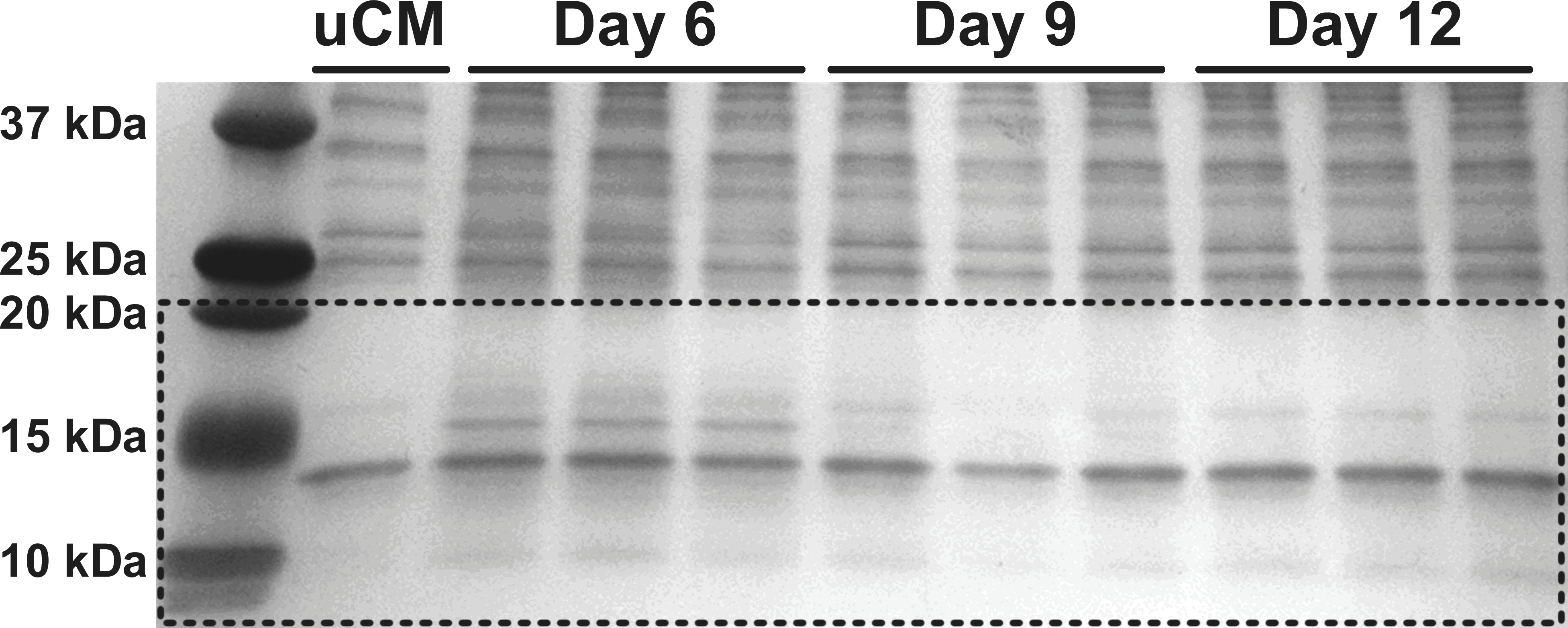


Additional file 1: Figure S1. EB-CM samples were analyzed using sodium dodecyl sulfate polyacrylamide gel electrophoresis (SDS-PAGE). Samples of equal volume were combined with 4X SDS-sample buffer consisting of 0.25 M Tris-HCl (pH 6.8), 8% SDS, 40% glycerol, 8% β-mercaptoethanol, and 0.02% bromophenol blue to reach a final concentration of 1X. These samples were then loaded into precast PAGEr 10-20% Tris-glycine gels (Lonza). The gels were run in 1X running buffer consisting of glycine, Tris base, and SDS for 2 hours at 125V. Gels were fixed and stained using Coomassie Blue. Images were then taken using a SONY DSC-W1 digital camera. 15x6mm (600 x 600 DPI).
