# Supplementary material for: Soluble factors secreted by differentiating embryonic stem cells stimulate exogenous cell proliferation and migration
Source: Stem Cell Res Ther. 2014 Feb 24;5(1):26. doi: 10.1186/scrt415 (PMC4055104; doi:10.1186/scrt415)
Supplement: Additional file 2 — Figure S2 showing the scratch wound migration assay: (A) phase image and (B) graph of cell migration. [file scrt415-S2.doc]

**Additional file 2: Figure S2.**


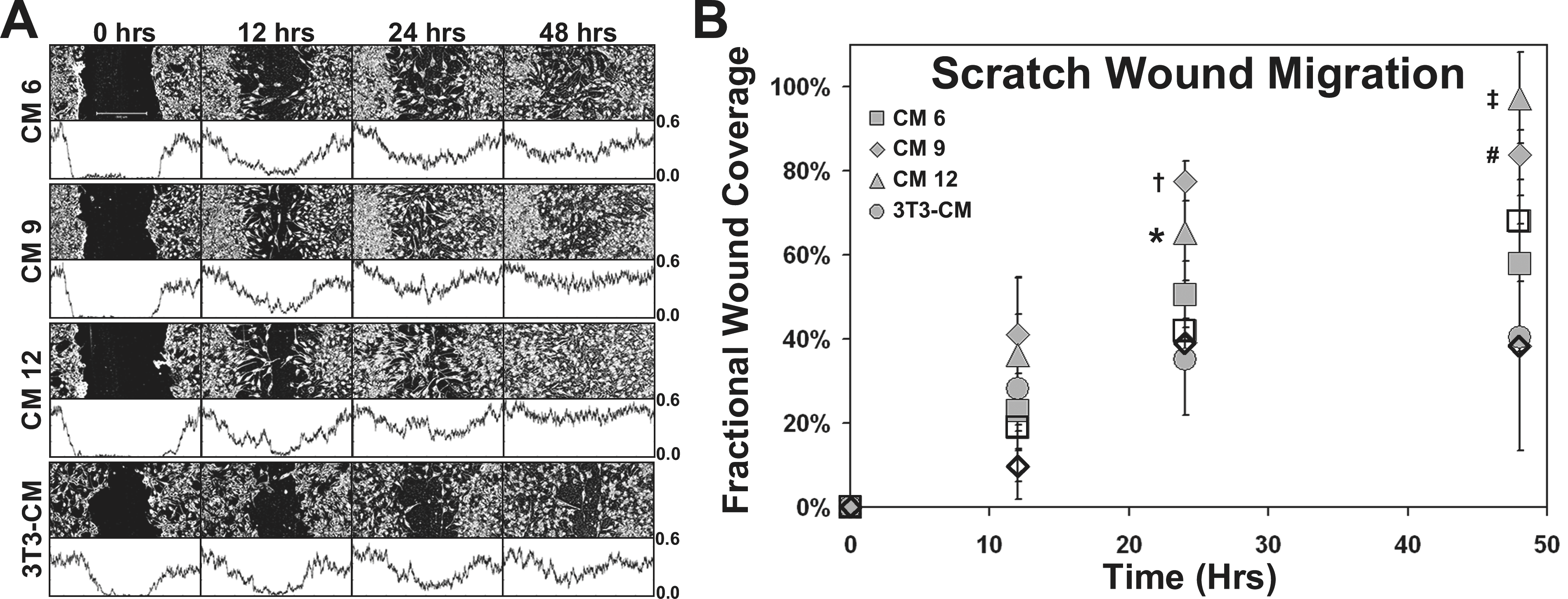


Additional file2: Figure S2. In a 6-well tissue culture plate, 3T3 fibroblast cultures were allowed to reach 80% confluence before being treated with serum-free 3T3 media containing 10 μg/mL mitomycin-C for 2 hours at 37^o^C in 5% CO_2_. Following treatment, cells were cultured overnight in 0.5% BGS 3T3 media. Next, the monolayer was scratched using the end of a 200 μL pipette tip, washed with dPBS to remove detached cell debris, and fed with 2 mL of EB-CM sample. At 0, 12, 24, and 48 hours media was removed and cells were rinsed with dPBS at which point phase images at 10X magnification were taken using a Nikon TE 2000 inverted microscope with a SpotFLEX digital camera (Diagnostic Instruments, Inc.). EB-CM was replaced following imaging. 14x5mm (600 x 600 DPI).
